# Using Free Adoptions to Reduce Crowding and Euthanasia at Cat Shelters: An Australian Case Study

**DOI:** 10.3390/ani7120092

**Published:** 2017-12-04

**Authors:** Heather M. Crawford, Joseph B. Fontaine, Michael C. Calver

**Affiliations:** Environmental and Conservation Sciences Cluster, School of Veterinary and Life Sciences, Murdoch University, Murdoch 6150, Australia; crawfh01@gmail.com (H.M.C.); j.fontaine@murdoch.edu.au (J.B.F.)

**Keywords:** Australia, cat, euthanasia, free adoption, husbandry, legislation, promotion, shelter

## Abstract

**Simple Summary:**

Waiving adoption fees to encourage adoptions and reduce euthanasias of healthy adult cats in crowded shelters is controversial because of concerns that people attracted to free adoptions may be less responsible owners. An extremely busy kitten season in 2015 left no shelter or foster vacancies for incoming cats at Western Australia’s largest cat shelter. Instead of euthanasing healthy cats, the shelter held a three day adoption-drive where cats ≥1 year were free. Public response to the event was extremely high (weekly adoptions increased >5-fold). Post-adoption surveys were carried out and results compared with surveys of cat adopters who paid normal-fees during non-promotional periods. No differences were found between free and normal-fee adopter demographics, cat demographics, cat fate post-adoption, incidence of medical and behavioural issues, and likelihood of attaching collars, registering with local councils or allowing cats to roam freely. Mixed-media promotion of the adoption-drive attracted more first-time adopters compared with normal-fee adopters. Overall, we found no evidence for adverse outcomes associated with free adoptions. Animal shelters should not be dissuaded from occasional free adoption-drives during overflow periods.

**Abstract:**

Many healthy adult cats are euthanised annually in shelters, and novel approaches are required to reduce euthanasia rates. Waiving adoption fees is one such approach. However, concerns that less responsible owners will be attracted to free events persist among welfare groups. We evaluated evidence for differences in cat fate, health, and adherence to husbandry legislation via a case-study of a free adoption-drive for cats ≥1 year at a Western Australian shelter. Post-adoption outcomes were compared between free adopters and a control group of normal-fee adopters. The free adoption-drive rehomed 137 cats, increasing average weekly adoptions by 533%. First-time adopters were a significantly larger portion of the free cohort, as a result of mixed-media promotions. Both adopter groups selected cats of similar age; sex and pelage. Post-adoption, both groups retained >90% cats, reporting near identical incidences of medical and behavioural problems. Adopters did not differ in legislative compliance regarding fitting collars, registering cats, or allowing cats to roam. The shelter reported satisfaction with the adoption-drive, because in addition to relieving crowding of healthy adults, adoption of full-fee kittens increased 381%. Overall, we found no evidence for adverse outcomes associated with free adoptions. Shelters should not be dissuaded from occasional free adoption-drives during overflow periods.

## 1. Introduction

Globally, the domestic cat (*Felis sylvestris catus*) is a popular companion animal, with 18% of UK households [1], 26% of European households [2], 35% of USA [3], 44% of New Zealand [4] and 29% of Australian households [5] owning cats. Popularity, and a continuous reproductive cycle with induced ovulation (~2–3 litters per year depending on age, health, number of daylight hours and latitude [6,7,8,9,10,11,12]), inevitably leads to uncontrolled breeding and abandonment of cats at welfare shelters. For example, from mid-2015 to mid-2016, cats comprised 40% of the total number of animals processed nationally by the Australian Royal Society for Prevention of Cruelty to Animals [13]; higher than dogs at 33%. The total number of cats euthanized over the same period was nearly three times that of dogs (16,205 cats vs. 5872 dogs). A similar situation occurs in the United States, where a survey of American shelters revealed for the fiscal year 2008–2009 the highest euthanasia rates for cats in a shelter was 71%, compared to 35% for dogs in the same facility [14].

While many cats entering shelters are euthanised due to illness, disease or behavioural issues [15,16], healthy adult cats are also euthanised due to shelter crowding or financial strain [14,16]. A few countries prohibit this by law (e.g., Brazil [17] and Czech Republic [16]), but that may create other problems such as crowding or refusal to admit animals to a full shelter. Compared with adult cats, kittens may be more expensive to care for. In addition to requiring multiple vaccinations and more frequent worming than adult cats, there is potential extra nutritional care and associated labour (e.g., feeding every 4 h for kittens that have not been weaned). However, kittens are easier to rehome and can attract higher adoption fees [15,18], so they may receive preferential treatment and housing over healthy or resident adult cats during times of crowding. High, on-going euthanasia rates also precipitate stress and poor morale of shelter staff, veterinarians and volunteers [19,20]. It is, therefore, in the best interests of both cats and staff that shelters minimise euthanasia rates.

To avoid euthanasia of older but otherwise adoptable cats, shelters may employ a range of strategies including refusing admission of cats during busy periods (e.g., ‘kitten seasons’), and outsourcing cats to other charities, management groups or foster caregivers. Alternatively, shelters may opt to hold adoption-drives wherein typical adoption fees are reduced or waived to achieve higher adult rehoming rates and relieve capacity pressures [21,22].

Waiving adoption fees is the most controversial option, because of concerns that people attracted to low-cost or free adoptions may be less responsible owners who subsequently neglect or rehome cats, or use them for nefarious purposes (e.g., kitten farming, dog baiting). However, in Maine, United States, whether or not adopters paid for their cat had no influence on their emotional attachment to their pet, or their opinion of the shelter [21]. Similarly, in Queensland, Australia, adopters who paid a discounted fee of $20 or regular fee ≥$99 for adult cats, did not differ in their retention of the cat at time of survey. Both groups also had similar self-rated attachment to- and satisfaction with, their cats. They did not differ in their intention to keep the cat, willingness to adopt from the shelter in the future and in caretaking behaviours shown towards the cat (e.g., frequency of petting, worming etc.). However, the authors acknowledged imprecision in the effect estimates in their statistical analyses [22].

Given the limited number of studies on the topics of discounted or free adoption-drives and concern over the strength of inference from prior studies, we sought to add further evidence to the literature via a case study of a free-adoption drive at a Western Australian cat shelter that focused on characteristics of adopters (age, gender, history of prior adoptions, source of information about the shelter), fate of cats (retained, deceased, returned to shelter, rehomed privately), compliance of owners with relevant legislation (Western Australian Cat Act 2011), and owner experiences with shelters. We also compared the data from the free-adoption drive with control data from normal-fee adoptions from the same shelter to see if paying adopters differed from free adopters on these points. Although the study was primarily descriptive, based on the limited available literature, we predicted that:

1. Cat demographics (age, sex and pelage) and fate (e.g., dead, returned) would not differ between the free and normal-fee adopters;
2. Human demographics (e.g., gender, distance from shelter, average income) would not differ among free and normal-fee adopters; and
3. Both groups would be equally compliant with the requirements of relevant Western Australian legislation.

Evidence consistent with these predictions would provide further support for shelter managers to proceed with greater confidence to use free adoptions in order to relieve accommodation and resource pressures during peak periods. In contrast, lack of support for our predictions would suggest reconsideration of free adoptions as a viable strategy for decreasing euthanasia.

## 2. Materials and Methods

### 2.1. Study Context

The Cat Haven shelter in Perth, Western Australia (WA), is the state’s largest recipient of feral, stray and surrendered cats, processing >4000 cats per year. These cats come mainly from across the 30 local government jurisdictions in Perth (~1.95 million people in 2016 [23]), but small numbers do come from outside the Perth metropolitan area. In accordance with the Australian Cat Action Plan for cat management ([24] developed by Getting2Zero), the Cat Haven has a long-term goal of zero euthanasia, excepting those animals which will never be rehomeable because of factors such as terminal illness or lack of socialisation. During 2014 and 2015 weekly euthanasias fluctuated seasonally, but for adult and mature cats were mainly in the range 2–22 and 0–21 for kittens. Despite having year-round discounted prices for cats over one year of age, an extremely busy 2014–2015 kitten season left the shelter stretched beyond capacity. A decision was made to trial a three-day adoption-drive where adult cats (≥1 year) would be given away for free.

The Cat Haven desexes and microchips all cats prior to adoption, so we focused on other facets of animal husbandry likely to vary among owners: fitting collars, council registration, and whether or not cats were permitted to roam freely. The Cat Haven provides all cat adopters with a booklet of information on basic cat behaviours and tips for assimilating them into new homes; possible illnesses, and worming and vaccination routines. The Cat Haven has an adoption process that includes verifying photographic identification and address, and a right to refuse service to uncooperative customers.

The legal context within Western Australia changed in 2011 with the passage of the Cat Act [25], which requires WA cat owners to ensure that by six months of age all cats are desexed, microchipped, wearing a collar and registered with the owner’s local municipal council. While the Cat Act (2011) does not mandate restricting cats to their owners’ property, we take the provision of fines, trapping and euthanasia of nuisance animals as an implication that owners should not permit their cats to roam. As such, the Cat Haven distributes leaflets on the WA Cat Act regulations and requirements for responsible cat ownership to adopters, and sends a notice of adoption to the adopter’s local council so that cat legislation may be monitored and enforced.

### 2.2. Data Collection

In February 2015, a week-long advertising campaign promoted the ‘free adult cat adoption-drive’ via Cat Haven’s website, social media pages, local newspapers, radio and television-news programmes. Free cats included ‘Adults’ (1–7 years old), and ‘Matures’ (>7 years). Kittens remained available for adoption at their regular year-round prices. Over the three day long-weekend (28th February–2nd March), 137 free cats were adopted (118 adult and 19 mature). Of these, the 100 cat adopters who had provided useable contact details were approached 6–12 months after adoption, using emails, postal letters, and telephone interviews (*n* = 50, 25, and 25 respectively) depending on personal details voluntarily provided at time of adoption.

A larger post-adoption study using the same survey was carried out between December 2014 and May 2015. A further 120 randomly-selected people (as the largest manageable sample size for our team) who adopted adult or mature cats for a ‘normal-fee’ ($50–$180) during this period, were contacted and their responses compared with those of free cat adopters. Subjects were selected using random number protocols. Normal-fee adopters were contacted 3–18 months post-adoption using emails, postal letters, and telephone interviews (*n* = 45, 50, and 25 respectively).

To gauge the Cat Haven’s perception of the event, we invited the CEO to comment on experiences over the weekend, and asked specifically about euthanasia and financial implications of the free adoption-drive. In order to assess how rates of adult/mature cat adoption over the free weekend differed from normal weeks of the year in 2015, the total weekly number of adoptions was plotted using the ggplot2 package within R version 3.1.1 [26,27]. The total weekly number of kitten adoptions was also plotted for comparison, as were adoptions over the course of 2014. Euthanasia data for the years 2014 and 2015 were provided by the Cat Haven as a context for assessing the potential effect of the free weekend on euthanasia rates.

### 2.3. Questionnaire Design and Owner Demographics

Surveys for owners adopting free or normal-fee cats were identical (Appendix A Table A1). Surveys focused on the husbandry and fate of adopted cats, the gender of the adopters, their adoption history with cat shelters and means of learning about the Cat Haven shelter. Demographic information for adopter suburbs was obtained from the Australian Bureau of Statistics ([23], see results), in preference to producing a longer, less user-friendly questionnaire if this information was requested directly from adopters.

### 2.4. Statistical Analyses

Differences in response rates between free and normal-fee adopters were assessed using the interface for comparing proportions in the online resource VassarStats [28]. Basic demographics of cats adopted for free or for payment were tabulated, noting sex, age in months, pelage, and fate after adoption. Similarly, the demographics of owners adopting cats (free vs. paid) were tabulated, noting gender, adoption history, knowledge of Cat Haven, and a range of characteristics of their suburb (e.g., distance from shelter, population density, mean salary [23]) as potential indicators of socio-economic status. Based on the people who responded, this resulted in a data matrix of 151 cats and 141 unique households. All subsequent statistical analyses of these variables used lme4 and ggplot2 packages within R version 3.1.1 [26,27,29].

To assess associations between adoption fee (free vs. paid), cat attributes (age category, sex, pelage, adoption fate), and adopter attributes (gender, adoption history with shelters, means of learning about Cat Haven), count data were assessed using two-tailed Fisher exact probability tests or chi-square contingency tables. For suburb-scale data, Student t-tests were used to compare suburb data (e.g., average income) with adoption fee. The sequential Bonferroni correction was applied to the t-test data to ensure a significance level of 0.05 across the full range of tests [30].

The two mandatory legislative variables (collars and registration) and whether cats roam beyond adopter residences, were all assessed using mixed-effect hierarchal models. The dependent variable in each case was binary (compliant or not compliant) and the predictors were whether the adoption was free or paid, age of the cat in months, cat sex, gender of the adopter, and whether or not they had adopted from a shelter before. We applied a mixed-model approach because 10 pairs of cats were adopted into the same households. Therefore, the household was treated as a random effect. Effects with *p*-values between 0.01 and 0.05 were considered as offering modest significance and effects with *p* < 0.01 as offering stronger evidence of a difference. Given the range of times post-adoption when questionnaires were administered, Fisher exact tests were used to assess any effect of number of months cats were owned at time of survey (<6 months, <12 months, <18 months) against compliance with collar, registration and roaming variables. Sequential Bonferroni corrections were applied because of the multiple tests.

### 2.5. Ethics Approval

This research was carried out in accordance with Murdoch University’s Human Ethics Approval 2014–099.

## 3. Results

### 3.1. Profile of Responses

Of the 100 free cat adopters surveyed, 69 responses were received (32 email; 18 letter; 19 telephone; 69% reply rate), representing 65 singles, and four pairs of cats (total *n* = 73; Table 1). Of 120 normal-fee cat adopter surveys, 72 responses were received (68 email; 4 telephone; 60% reply rate), representing 66 single and 6 pairs of cats (total *n* = 78; Table 1). The response rates of the two groups did not differ significantly (two-tailed comparison of proportions, *z* = 1.39, *p* = 0.16).

### 3.2. Cat Demographics

The demographics of cats did not differ between free and normal-fee adoptions (Table 1). Nearly two thirds of the adoptions in each group were female cats, with no significant association between adoption fee and cat sex (two-tailed Fisher exact test *p* = 0.86), age category of the cats (two-tailed Fisher exact test *p* = 0.11), or pelage (chi-squared contingency table χ_1, 5_ = 6.39, *p* = 0.27). The fate of cats was also similar between the two groups (Table 1), using the categories of ‘Alive and retained by original adopter’ and pooled fewer frequencies of ‘Dead’, ‘Ran Away’, ‘Rehomed’ and Returned’ (two-tailed Fisher exact test *p* = 0.48). No returned cat had a history of failed adoptions. Adopters reported near-identical incidences of medical and behavioural problems for free and normal-fee cats (Table 2).

### 3.3. Adopter Profiles

The gender profiles of free and normal-fee cat adopters were very similar (Table 3), with more female than male adopters in both samples (two-tailed Fisher exact test *p* = 0.82). More free- than normal-fee cat adopters were adopting from a shelter for the first time (Table 3; two-tailed Fisher exact test *p* = 0.04). There was also a difference in the way in which adopters learned about the existence of the Cat Haven shelter (Table 3; word-of-mouth, media and pooled miscellaneous categories, two-tailed Fisher exact test *p* < 0.001), with most free cat adopters learning of the Cat Haven through promotion of the adoption-drive using various media (e.g., radio, social media). Normal-fee cat adopters mainly learned about the Cat Haven through word-of-mouth. After sequential Bonferroni correction, Student t-tests revealed no significant differences between adoption fee and any characteristics of suburbs where owners resided (Table 4).

### 3.4. Compliance with Regulations

Survey responses were tallied to assess compliance with three responsible cat ownership variables under state legislation (two mandatory, one implied; Table 5).

Two-tailed Fisher exact tests revealed there was no difference between free and normal-fee adopters’ adherence to collar (*p* > 0.99), registration (*p* = 0.14) or roaming (*p* = 0.87) legislation. After sequential Bonferroni correction, the number of months cats had been owned when surveyed did not affect whether cats wore collars (free *p* > 0.99, normal-fee *p* = 0.10) or were registered (free *p* > 0.99, normal-fee *p* = 0.24). Months owned at survey also did not affect whether free cats (*p* = 0.24), or normal-fee cats (*p* = 0.02) could roam freely. Mixed-effect hierarchal models detected only one modest association between cat predictors and husbandry response variables, which was a greater likelihood for male adopters to allow cats to roam (Table 6).

### 3.5. Shelter Perception of the Free Adult Cat Adoption-Drive

**Figure 1:**
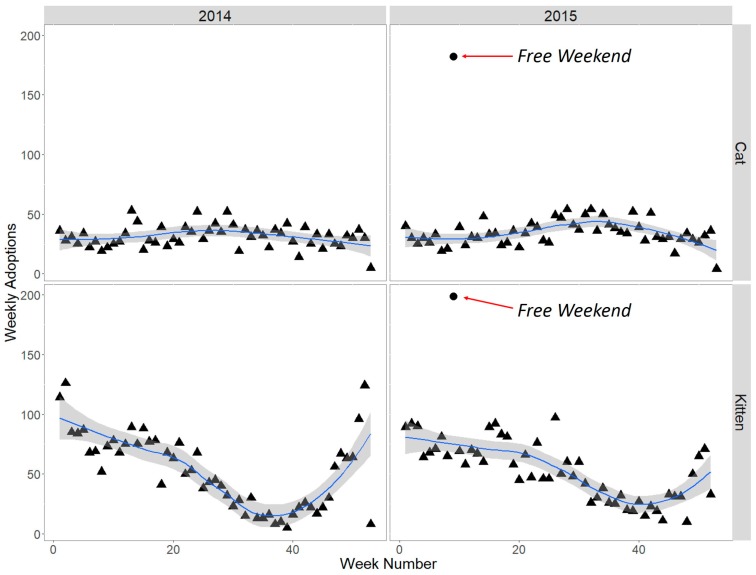
Weekly cat and kitten adoptions 2014–2015 at the Cat Haven, WA, with a loess smoother and 95% confidence envelope. The free weekend dot indicates the number of adult and mature cats or kittens adopted during the entire week that included the adoption-drive. Free weekends increased cat adoptions by 533% and kitten adoptions by 381% relative to 2015 weekly averages.

Public response to the free adult cat adoption-drive was so high that all shelter-housed cats were adopted before close of the first day, and over the course of the weekend healthy adults in foster-care were recalled to the shelter to fill newly created vacancies. In total, 137 healthy adult and mature cats were adopted during the free weekend and thereby spared euthanasia. During the week including the free weekend in 2015, 182 adult and mature cats were adopted. In comparison with the mean adoptions of adult and mature cats for all non-promotional weeks in 2015 (34.11 cats), the free adoption-drive increased weekly total cat adoptions by approximately 533% (Figure 1). Adoptions from the same week in 2014 totalled 22 cats, considerably less than the 182 in 2015.

The free adult adoption-drive also substantially increased adoptions of full-fee kittens, with 154 kittens adopted during the free weekend. During the week including the free weekend in 2015, 199 kittens were adopted. In comparison with the mean adoptions of kittens for all non-promotional weeks in 2015 (52.25 kittens), the free adoption-drive increased weekly total kitten adoptions by approximately 381% (Figure 1). Adoptions from the same week in 2014 totalled 73 kittens, considerably less than the 199 in 2015. Adoption of 154 normal-fee kittens during the free weekend created space for new incoming cats and, despite waiving adult and mature cat adoption fees, the Cat Haven CEO R. Robinson states that “More revenue was generated in three days than is normally made in three weeks from purchases of food, accessories and full-fee kittens.” 

**Figure 2:**
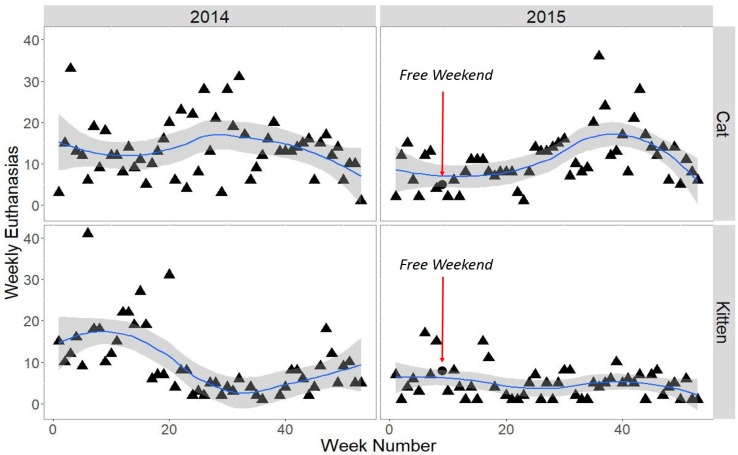
Weekly euthanasia rates for cats and kittens 2014–2015 at the Cat Haven, WA, with a loess smoother and 95% confidence envelope. The free weekend dot indicates the number of adult and mature cats or kittens euthanised during the entire week that included the adoption-drive.

During 2014 and 2015 weekly rates of euthanasia (Figure 2) included no healthy adult cats, in accordance with Cat Haven’s long-term goal of zero euthanasia of healthy animals. The main reasons for euthanasia were health (e.g., feline calici virus) or behaviour-related (e.g., feral). Cat Haven CEO R. Robinson commented: “Faced with so many cats (in 2015) we couldn’t rehome due to kittens being available, we had to take radical steps. Being an open admission shelter, we simply couldn’t continue to keep taking cats in, without getting them out. We had two choices, either give the cats away for free, or euthanize them, Euthanasia was not an option as we felt it was unethical to put healthy and friendly cats to sleep.”

## 4. Discussion

In keeping with our predictions, demographics of cats adopted did not differ between free and normal-fee adoptions. Both groups adopted cats of similar age, gender and pelage. Comparison of the fate of cats adopted for free or for a fee, revealed that the majority of cats were alive and retained by their original adopters (93% for free cats, 96% for normal-fee cats). These findings closely parallel those of an Australian study (state of Queensland [22]) which found that 6–12 months after adoption 91% of $20 cats (*n* = 126) and 90% of ≥$99 (*n* = 17) cats were alive and retained by the original adopter.

Post-adoption, the incidences of medical and behavioural problems were also nearly identical, leaving 77% of both free- and normal-fee cats alive and well. Treatable medical problems developed in only 7% of free, and 6.5% of normal-fee cats (e.g., diarrhoea, skin rash, haematuria), with 80% of unwell cats receiving veterinary treatment, regardless of adoption fee. Including the three cats returned to the Cat Haven, only 16.5% of free, and 18% of normal-fee cats were reported as having any enduring behavioural problems, with free cats mainly shy or hiding in fear (33%), and normal-fee cats mainly aggressive towards owners or destructive (43%). Problem behaviours can be common in cats [31,32], and cats are frequently surrendered or returned to shelters if they do not rapidly adjust to their new home, or display antisocial, destructive or inappropriate elimination behaviours [33,34,35,36]. For example, over the course of one year, 33% of cat owners cited behaviour problems as reasons for surrendering their cats to 12 animal shelters in the USA (*n* = 2168 [36]). The near-identical low incidence of reported behavioural problems in free and normal-fee cats is consistent with owners in the two groups perceiving the behaviour of their cats similarly, some of which may be natural behaviour that needs appropriate redirection [37].

Overall, there is no evidence that adoption fee influences the selection of cats or their fate with their new owners. Perception of behavioural problems, which is an acknowledged risk for return of adopted cats to a shelter, was also similar between the two groups and likely contributed to the similar fates of cats in both groups.

### 4.1. Do Free Adoption-Drives Attract a Different Group of Adopters?

The care that pet cats receive from owners may be influenced by owner features such as age, income, gender, education, previous experience owning pets, presence of children, and number of emotional bonds with friends [38,39]. However, we found that both free and normal-fee adopters were similar across 12 different demographic variables constructed from ABS suburb-scale data. Where the two groups did differ was in their experience adopting from shelters, with more first-time adopters visiting the Cat Haven during the free adult adoption-drive. It is perhaps unsurprising then that the groups also differed in how they initially learned of the Cat Haven’s existence, with free adopters learning about the shelter from media promotions of the free adoption-drive, and normal-fee adopters learning through word-of-mouth. Given that pet cats have extended life expectancies (~15 years for indoor cats and 10 years for outdoor cats [40]), and that this may limit the number of experienced cat owners who are available to adopt, attracting first-time adopters must be a priority for shelters wishing to decrease euthanasia of healthy cats.

### 4.2. Does Adoption Fee Influence Legislative Compliance?

Adopters of free and normal-fee cats did not significantly differ in their legislative compliance. Both groups were as likely to place collars on cats (69% free vs. 65% normal-fee), register them with local municipal councils (75% vs. 81%), and allow cats to roam outside and beyond their residence boundary (53% vs. 55%). The high compliance with registration may reflect Cat Haven’s legal responsibility to notify local councils with details of adoptions, as well as the shelter’s efforts to communicate the responsibilities of all cat owners. Registration is not free (fees vary between municipalities), so cost might be a factor in determining whether or not owners register their animals. However, there was no statistically significant difference in the proportion of cats registered between free and normal-fee adopters.

All Australian states and the Australian Capital Territory have legislation governing aspects of cat ownership, but there is little data on compliance and much of that suggests that compliance is variable and low. For example, in 2001 approximately 500,000 dogs and cats were microchipped in New South Wales under legislative requirements, but only 200,000 animals were registered [41]. In one Victorian municipality only 15% of households registered a cat, substantially below the national cat ownership estimate of 26% of households [42]. In relation to confinement of pet cats, many owners appear to make a decision about husbandry based on what they believe is best for their cat’s welfare, and do not always comply with legislation that they feel compromises welfare [43]. Welfare considerations may also apply when owners choose whether or not fit a collar, because of perceptions that collars are hazardous to cats despite data showing that the risk is low and the benefits of simple identification high [44,45]. Overall, decisions about compliance are complex and the modest compliance noted in our samples may or may not be representative of national trends.

### 4.3. Why Are Free Adoptions Good for Cats?

Preventing euthanasia of healthy adult cats is an obvious, immediate positive outcome of reducing crowding by offering occasional free adult cat adoption-drives [15,46]. Moving adult and mature cats through shelter systems quickly also benefits general welfare by minimising exposure to diseases such as upper respiratory tract infections or ringworm [47,48,49,50,51], and to unpredictable, sustained or novel stimuli such as handing by visitors [52], that can cause stress-related urinary tract infections [53], and behavioural issues such as abnormal eating or grooming [54]; elimination [55]; and hypervigilance [53]. Furthermore, because many factors influence the likelihood of adoption including cat sex (e.g., males preferred [15]), appearance (e.g., rare breeds preferred, prejudices against darker coat patterns [18,56,57]), and perceptions of cat personality (e.g., playfulness and willingness to interact with people increases adoptions [58,59,60]); in a free adoption scenario where cats are being rapidly rehomed, the less popular animals may have a better chance of selection.

Pet cats surrendered to shelters have higher stress scores than stray cats, and this can negatively affect their health, length of stay and increase chances of euthanasia [61]. If cats enter an adoption-surrender cycle they may become traumatised and deemed unsuitable for rehoming. If free cat adoption-drives are used in times of crowding (or in anticipation of them) and are accompanied by rigorous pre-adoption counselling (as practised by the Cat Haven in this study) and supported by post-adoption advice or follow-up, then there is no reason to predict that free adult cats will be surrendered more readily than those sold for a fee.

### 4.4. Why Are Free Adoptions Good for Shelters?

Rehoming adult cats will reduce euthanasia rates and the associated risk of staff suffering ‘trauma fatigue’ [19,20]. Foster carers, who are often a pivotal resource relied on by animal shelters to help deal with burgeoning numbers of unwanted and juvenile animals [62,63], are also relieved when all surrendered animals can be accommodated within the shelter. Overall, shelters offering free adoptions will save the cost of long-term housing and provision of environmental enrichment for animals with lower adoption potential. Resources may then be redirected towards costs of caring for kittens or public education programs that may reduce abandonment of cats (e.g., increasing community desexing rates of cats). Furthermore, Cat Haven CEO R. Robinson noted “the weekend was a remarkable success. Adopters made generous donations, and bought quality food, and accessories for the cats they adopted.”

Free adoption-drives afford shelters with further opportunities to educate new, and experienced adopters, prior to point-of-sale. This education in the form of pre-adoption counselling, is crucial to breaking adoption-return cycles and reducing euthanasia. While many shelters routinely provide take-home leaflet information about cat behaviour, disease and responsible ownership, shelters are often unable to counsel potential adopters about their motivations for adopting, and what pet would be most suitable for their lifestyle. Yet, adoptions frequently fail as a result of unrealistic owner expectations about the role a cat will play in the family and of its temperament [64,65,66]. According to Podberscek [67] (p. 368), “Sometimes simple education about why an animal behaves in a certain way is enough to make the animal more acceptable in the eyes of the owner.” Therefore, offering pre-adoption counselling at all times may benefit shelters, cats and adopters in a number of ways. Firstly, new and experienced adopters will benefit equally from being matched with the best possible cat candidate for adoption, which should translate into faster acclimation to new homes, less stress for owners and fewer returned animals. Secondly, whether or not an adoption fee is paid, pre-adoption counselling for both new and experienced adopters will provide shelter staff with opportunity to assess the expectations of people adopting cats.

### 4.5. Limitations of the Study

This paper reports a single case study, which may not be representative of other shelters in other jurisdictions. However, it has value in adding data to an important question where there are few other studies, which also share the limitation of being geographically restricted [21,22]. Furthermore, this study adds the unique element of assessing legislative compliance to complement existing work on attachment levels and caring practices of owners [21], or outcomes for adopted cats [22]. While sample sizes for cats adopted were modest in our study (151), this is also true of existing studies (173 cats in [21], and up to 248 adult cats in [22]. The value comes from multiple studies contributing to different aspects of the outcomes of free adoptions, which all reach a similar conclusion that there were no serious negative outcomes for cats or participating shelters.

## 5. Conclusions

The Cat Haven, in common with many shelters globally, seeks to rehome unwanted cats and minimise or eliminate euthanasia of healthy cats. Owners who adopted free cats did not differ from fee paying adopters in demographics or compliance with husbandry legislation. Free cat adoption-drives are likely to attract first-time cat adopters, especially if shelters use a variety of media. This will help deal with issues of saturation among cat owners. Animal shelters should not be dissuaded from free adoption promotions during times of crowding, and may even wish to hold one in anticipation of a high demand for housing. Although these conclusions apply to one local shelter, they are also consistent with the limited literature on the topic of free and discounted adoptions [21,22].

## Figures and Tables

**Table 1:** Demographics of 73 free cats adopted (4 pairs) and 78 normal-fee cats (6 pairs) adopted from the Cat Haven shelter, WA, in 2013–2015.

Cat Demographics	Free Cats *n* = 73 (%)	Normal-Fee Cats *n* = 78 (%)
Cat Ages		
Age range	1–16 years	1–14 years
Age mean	4 years	3 years
Cat Sex		
TOTAL females	52 (71)	57 (73)
Female adults	46 (63)	54 (69)
Female matures	6 (8)	3 (4)
TOTAL males	21 (29)	21 (27)
Male adults	16 (22)	19 (24)
Male matures	5 (7)	2 (3)
Pelage Varieties		
Blacks and Browns	9 (12)	6 (8)
Calicos and Tortoiseshells	8 (11)	17 (22)
Brindles and Gingers	9 (12)	4 (5)
Greys and Whites	9 (12)	13 (17)
Piebalds	12 (16)	13 (17)
Tabbies	26 (36)	25 (32)
Cat Fate		
Alive and Retained by original adopter	68 (93)	75 (96)
^1^ Dead	3 (4)	0 (0)
^2^ Returned to shelter	0 (0)	3 (4)
^3^ Rehomed privately	1 (1)	0 (0)
^4^ Ran away	1 (1)	0 (0)

^1^ One cat died of cancer, one of liver disease and the third cat was hit by a car two weeks after adoption. ^2^ One mother-son pair of cats were returned to Cat Haven because of fighting with other cats in the household. The third cat was maladjusted to indoor-only living. ^3^ One cat developed a bond with a relative of the original adopter and was permanently rehomed with the relative. ^4^ Indoor-only cat did not return home after escaping through a door accidentally left open.

**Table 2:** Incidence of medical and behavioural problems in 73 free, and 78 normal-fee cats, as reported by WA adopters surveyed in 2013–2015.

Reported Cat Problems	Free Cats *n* = 73 (%)	Normal-Fee Cats *n* = 78 (%)
No medical or behavioural problems	56 (77)	60 (77)
Medical problems		
No medical problems	68 (93)	73 (93.5)
Skin rash/Systemic infection	5 (7)	4 (5)
Other	0 (0)	1 (1.5)
Number receiving veterinary treatment	4 (80)	4 (80)
Behavioural problems		
No behavioural problems	61 (83.5)	64 (82)
Number with ≥1 behavioural problems	12 (16.5)	14 (18)
Shy/Scared	4 (33)	3 (21.5)
Aggressive towards owners/Destructive	2 (16.5)	6 (43)
Fights with other pets	4 (33)	5 (36)
Toileting issues	3 (25)	0 (0)
Unhappy as inside-only cat	0 (0)	2 (14)
No medical or behavioural problems	56 (77)	60 (77)

**Table 3:** Survey responses of free and normal-fee cat adopters including gender, previous adoption history with cat shelters, and how adopters originally learned about the Cat Haven shelter, WA. Over the free adoption-drive weekend, four survey respondents adopted a pair of cats; and six pairs of normal-fee cats were adopted during the normal non-promotional period.

Variable	Responses	Free Adopters *n* = 69 (%)	Normal-Fee Adopters *n* = 72 (%)
Owner gender	Female	59 (86)	60 (83)
	Male	10 (14)	12 (17)
Adoption history	Never adopted from a shelter	57 (83)	46 (64)
	Previously adopted from a shelter	10 (14)	20 (28)
	Unknown	2 (3)	6 (8)
Information source	Media	40 (58)	12 (17)
	Word-of-mouth	21 (30)	38 (53)
	Miscellaneous	2 (3)	9 (12)
	Unknown	6 (9)	13 (18)

**Table 4:** Student *t*-test results for difference between 12 Western Australian suburb statistics [23] and cat adoption fee. No result is significant after sequential Bonferroni correction.

Suburb Parameters	*t*-Test Value	df	*p*-Value
Distance from shelter (km)	1.41	129	0.16
Number of people/km^2^	−2.29	148	0.04
Average number of people/household	1.00	144	0.32
Median age of people in suburb	−0.65	138	0.52
People with language other than English (%)	0.25	142	0.80
Average personal salary	−0.09	135	0.93
Total suburb income (millions $)	−0.49	149	0.63
Population with post-school qualification (% >15 years age)	−1.08	143	0.28
Unemployment rate	0.46	146	0.64
Working population (% 15–64 years age)	−0.69	126	0.49
Number of births	0.65	146	0.51
Number of deaths	−0.35	149	0.72

**Table 5:** Compliance of people adopting free and normal-fee cats with three variables of responsible cat ownership under WA legislation [25]. Wearing collars and registering cats with local councils are mandatory. Restricting cats to private property is recommended.

Variable	Responses	Free Cats *n* = 73 (%)	Normal-Fee Cats *n* = 78 (%)
Collar	Wears collar	50 (69)	51 (65)
	Does not wear collar	22 (30)	22 (28)
	Unknown	1 (1)	5 (6)
Registration	Registered with council	55 (75)	63 (81)
	Not registered with council	17 (23)	10 (13)
	Unknown	1 (1)	5 (6)
Roaming	Allowed to roam freely	39 (53)	43 (55)
	Not allowed to roam freely	34 (47)	34 (44)
	Unknown	0 (0)	1 (1)

**Table 6:** Mixed-effect hierarchal modelling used to assess relationship between free and normal-fee adopter husbandry response variables and adopted cat predictors. Effects with *p*-values between 0.01 and 0.05 were considered as offering modest significance and effects with *p* < 0.01 as offering stronger evidence of a difference.

Husbandry Variables	Cat Predictors	Estimate ± Std Error	*p*-Value	Interpretation of Husbandry vs. Adoption Fee
Cat wears collar	Free vs. normal fee	−0.006 ± 0.41	0.99	Likelihood of owner placing collar on cat does not vary with adoption fee
	Owner gender	0.20 ± 0.56	0.72	
	Previous adoption history	0.47 ± 0.51	0.35	
	Cat age in months	−0.20 ± 0.19	0.30	
	Cat sex	0.67 ± 0.49	0.17	
Cat is registered	Free vs. normal fee	0.71 ± 0.49	0.15	Likelihood of owner registering cat with local council does not vary with adoption fee
	Owner gender	0.38 ± 0.69	0.58	
	Previous adoption history	−0.58 ± 0.54	0.28	
	Cat age in months	−0.38 ± 0.20	0.06	
	Cat sex	0.91 ± 0.61	0.13	
Cat is allowed to roam	Free vs. normal fee	−0.26 ± 0.46	0.57	Likelihood of owner restricting cat to residence property does not vary with adoption fee
	Owner gender	−1.33 ± 0.67	0.05	
	Previous adoption history	−0.03 ± 0.54	0.95	
	Cat age in months	−0.03 ± 0.22	0.87	
	Cat sex	−0.15 ± 0.48	0.75	

## Appendix A

**Table A1:** Post-adoption surveys using email, letter and telephone questionnaires were identical for people who adopted cats for free, or for a normal-fee.

(1) OWNER DETAILS	(2) CAT DETAILS
Name and Surname	Number of cats adopted
Gender	Cat name
Address	Cat sex
Contact details	Approximate age when adopted
(3) SHELTER EXPERIENCE	(4) CAT HUSBANDRY
How did you come to learn of Cat Haven?	Does your cat wear a collar? Why/why not?
Have you previously adopted from shelters? If yes how many years ago?	Is your cat registered with your local municipal council? Why/why not?
Was the information provided at time of adoption and the take-home-info-pack of use to you? If yes how?	Is your cat given access to the outdoors? Why/why not?
What price did you pay for the cat?	If yes at what time and how often does your cat have access to the outdoors?
In your view can the Cat Haven improve anything?	
